# The Diverse Roles of Heme Oxygenase-1 in Tumor Progression

**DOI:** 10.3389/fimmu.2021.658315

**Published:** 2021-03-31

**Authors:** Kim Ngan Luu Hoang, Joanne E. Anstee, James N. Arnold

**Affiliations:** Faculty of Life Sciences and Medicine, School of Cancer and Pharmaceutical Sciences, King’s College London, London, United Kingdom

**Keywords:** heme oxygenase-1 (HO-1), cancer, cytoprotection, tumor immunology, angiogenesis, tumor associated macrophages (TAMs), metastasis

## Abstract

Heme oxygenase-1 (HO-1) is an inducible intracellular enzyme that is expressed in response to a variety of stimuli to degrade heme, which generates the biologically active catabolites carbon monoxide (CO), biliverdin and ferrous iron (Fe^2+^). HO-1 is expressed across a range of cancers and has been demonstrated to promote tumor progression through a variety of mechanisms. HO-1 can be expressed in a variety of cells within the tumor microenvironment (TME), including both the malignant tumor cells as well as stromal cell populations such as macrophages, dendritic cells and regulatory T-cells. Intrinsically to the cell, HO-1 activity provides antioxidant, anti-apoptotic and cytoprotective effects via its catabolites as well as clearing toxic intracellular heme. However, the catabolites of heme degradation can also diffuse outside of the cell to extrinsically modulate the wider TME, influencing cellular functionality and biological processes which promote tumor progression, such as facilitating angiogenesis and metastasis, as well as promoting anti-inflammation and immune suppression. Pharmacological inhibition of HO-1 has been demonstrated to be a promising therapeutic approach to promote anti-tumor immune responses and inhibit metastasis. However, these biological functions might be context, TME and cell type-dependent as there is also conflicting reports for HO-1 activity facilitating anti-tumoral processes. This review will consider our current understanding of the role of HO-1 in cancer progression and as a therapeutic target in cancer.

## Introduction

The heme oxygenase (HO) family of enzymes serve as the rate-limiting step in the degradation of heme which is released by dying cells and yields the biologically active catabolites biliverdin, ferrous iron (Fe^2+^) and carbon monoxide (CO) (1). The HO-1 isoform has been demonstrated to be expressed in a wide variety of cancers and has become implicated in a diverse range of biological processes which can be exploited by the tumor to facilitate disease progression and metastasis. The pivotal role of HO-1 in tumoral immune suppression in some preclinical models suggests it could represent an immunotherapy target and innate immune checkpoint (2). There are two isoforms of HO expressed in humans and mice; HO-1 which is inducible (3), and HO-2 which is expressed at basal levels in all cells (4). There is also a third non-catalytically active isoform, HO-3, that has been identified in rats (5). The HO-1 isoform is induced by a variety of external stimuli including; oxidative stress, cytokines and prostaglandins (6). Heme is also a potent inducer of HO-1 expression and acts as a cytoprotective measure against the pro-oxidant properties of free-form heme (7). As such, it is not surprising that HO-1 is expressed in a variety of cancers including; bladder (8), breast (2), colorectal (9), glioblastoma (10), head and neck (11), leukemia (12), lung (13), melanoma (14), neuroblastoma (15), prostate (16) and renal (17) cancer. HO-1 in the TME has also been associated with poor prognosis for patients (2, 15, 18). HO-1 is a 32kDa protein that predominantly localizes to the endoplasmic reticulum (ER) (19, 20) but can also localize to the caveolae (21), mitochondria (22), and nucleus (23). HO-1 has also been detected in the plasma and its concentration has been demonstrated to be elevated in prostate cancer (24). At the cellular level, HO-1 has most frequently been reported to be expressed by tumor cells (12, 25) and tumor associated macrophages (TAMs) (2, 26, 27) within the TME. However, HO-1 can also be expressed by endothelial cells (28), dendritic cells (DCs) (29, 30) and regulatory T-cells (Tregs) (31, 32). This review will discuss the current knowledge and controversies on the biological roles of HO-1 in the TME and as a therapeutic target in cancer.

## Biochemical properties of HO-1

**Figure 1:**
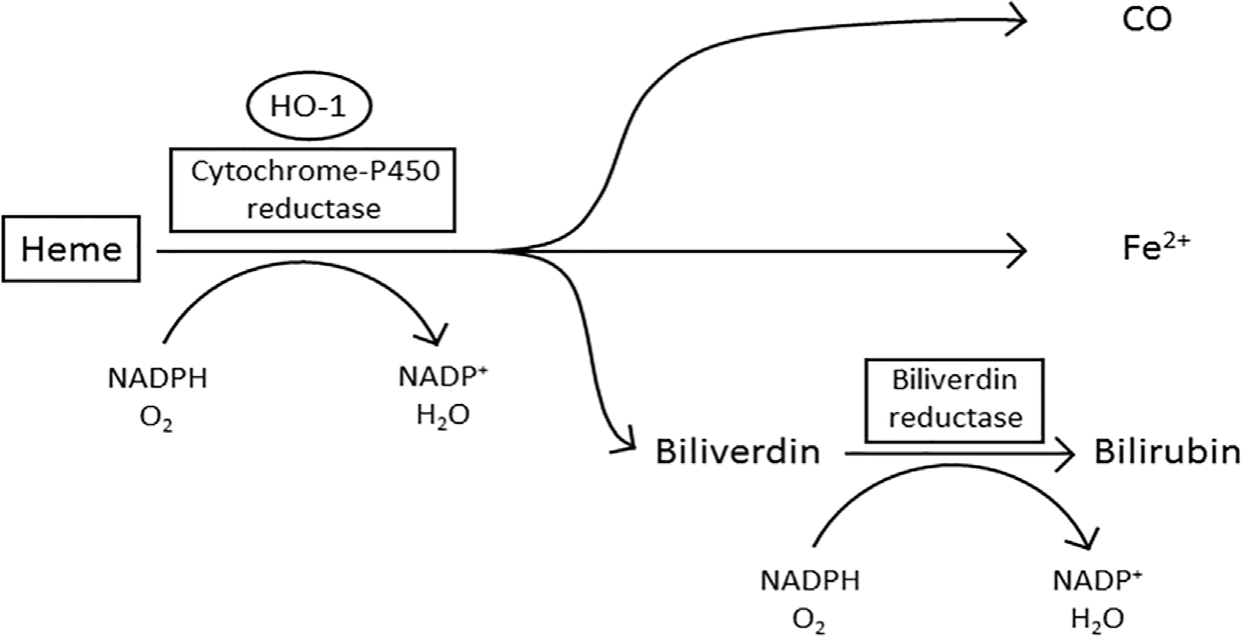
The oxidative degradation of heme by HO-1. In the first step of heme degradation, the ER membrane bound HO-1 interacts with the electron donor NADPH-cytochrome P450 reductase, and an oxygen molecule. The complex degrades heme to biliverdin, carbon monoxide (CO), and a ferrous iron (Fe^2+^). NADPH-biliverdin reductase competitively binds to HO-1 to reduce biliverdin to bilirubin, by using NADPH as an electron donor (1, 33).

Enzymatically active HO-1 predominantly localizes to the ER within the cell where it forms the microsomal heme oxygenase system (19, 21) (**Figure 1**). Heme is pro-oxidative and cytotoxic, due to the protein being involved in lipid peroxidation and catalyzing the production of free radicals (34, 35). Furthermore, heme can also trigger cell damage and apoptosis by inhibiting the proteasome and causing mitochondrial dysfunction (36–38). In the first stage of oxidative heme degradation, HO-1 forms a complex with heme and NADPH-cytochrome-P450 reductase (39) (**Figure 1**). NADPH acts as the electron donor while molecular oxygen binds to the complex, yielding CO, chelatable Fe^2+^ and biliverdin (40). Subsequently, biliverdin is converted to bilirubin by the enzyme NADPH-biliverdin reductase (BVR) using NADPH (1, 33, 41). The catabolites of heme degradation are biologically active and go on to elicit a variety of cell intrinsic and extrinsic effects (42). A truncated variant of HO-1 has also been identified in malignant cells which localizes to the nucleus and appears to be associated with a cellular response to hypoxia (23, 43). Nuclear localization of HO-1 requires the proteolytic cleavage of the C-terminal 23 amino acids of the protein and then chromosomal maintenance-1 (CRM1)-mediated nuclear-cytoplasmic shuttling to the nucleus (23). The truncated HO-1 variant, although not catalytically active, appears to be a response specifically of malignant cells rather than the non-malignant stromal cells within the TME (16). Within the nucleus the truncated HO-1 variant has been demonstrated to play a role in transcriptional regulation, down-regulating NF-κB and SP-1 DNA binding activity, while up-regulating oxidant-responsive transcription factors, such as activator protein-1 (AP-1), AP-2, Brn-3 and core-binding factor (23). The biological importance of truncated HO-1 in relation to its impact on cancer progression still needs further investigation, however it has now been observed in a range of cancers, but most well characterized in prostate cancer, where it has been associated with disease progression (11, 44). The non-catalytic function of HO-1 provides an intriguing additional layer of functionality to the protein.

## The Sources of HO-1 in the TME

In the TME, HO-1 can be expressed by both the malignant tumor cells and stroma (2, 27, 45). Several stromal cell populations have been described to express HO-1 (45). TAMs, which and are a prevalent stromal cell type in the TME, and are a major stromal source of HO-1 in both murine models (2, 26, 27) and human disease (2, 27). In some TMEs, TAMs represent the major tumoral source of HO-1 (2, 14, 26, 27, 46). TAMs are a highly plastic stromal cell type which are capable of responding to the TME to adopt a variety of distinct phenotypes, creating a spectrum of possible polarization pro- and anti-inflammatory phenotypes that differ in their cytokine profiles and gene expression (47–50). HO-1 is not a pan-TAM marker per se, and is instead selectively up-regulated by a subset of these cells, suggesting its expression is restricted to certain polarization states (2, 14, 26, 27, 46, 51, 52). HO-1 has been associated with TAMs with a polarization program similar to the ‘M2’ tumor-promoting macrophage phenotype, and there is also evidence to suggest that HO-1 may play a direct role in skewing the macrophage program (53). In further support of this, inhibition of HO-1 activity in macrophages has been demonstrated *in vitro* to revert their phenotype to a more inflammatory state, marked by an increase in inducible nitric oxide synthase (iNOS) expression (51). A subset of HO-1 expressing TAMs can be identified, in certain TMEs, to co-express fibroblast activation protein alpha (FAP), a surface protease associated with fibroblast populations (26, 27, 54). In subcutaneous Lewis lung carcinoma (LL2) tumors, the FAP^+^ TAM subset accounted for 10% of the total TAM population and represented the major source of HO-1 in those tumors (26). FAP^+^ TAMs have also been identified in human breast cancer (55) and this subset also expresses HO-1 (27). In orthotopic 4T1 tumors a FAP^+^ HO-1^+^ TAM subset was also identified and, in this model, was associated with a macrophage polarization response to IL-6 in a TME rich in collagen (the substrate for FAP’s protease activity) (27). 4T1 tumors displayed a ‘healing wound’-like TME and, as such, it was not surprising that FAP^+^ HO-1^+^ macrophages were also present in the granulation tissue of a cutaneous healing wound (27). This observation highlights how tumors exploit TAM polarization from fundamental biological responses of these cells. Interestingly, the relationship between TAMs and HO-1 does not always promote tumor progression. In an A549 lung carcinoma model the presence of CD86^+^ myeloid cells, which are regarded as a pro-inflammatory M1-like macrophage phenotype, were required to mediate the anti-tumor growth effect of exogenously administered CO to tumor bearing mice (56).

DCs, which represent a professional antigen presenting cell type that can reside in the TME have been observed to express HO-1. HO-1 expression by DCs can also influence the activity of these cells, suppressing their immunogenicity and antigen presentation capabilities (29, 30). The immunomodulatory CD4^+^ CD25^+^ Foxp3^+^ Treg population has also been described to express HO-1 in humans (31, 32) but not mice (57), and may be directly linked to the Foxp3-regulated gene expression program in humans (58). As the catabolites of heme degradation can diffuse extracellularly to influence non-HO-1 expressing cells, the importance of the specific cell-type expressing HO-1 in relation to prognosis remains to be fully resolved, where location and prevalence could be the most important variables to consider. The widespread expression of HO-1 in cancer also highlights the fundamental relationship between the enzyme and the disease.

## Signals In The TME for *HMOX1* Gene expression

**Figure 2:**
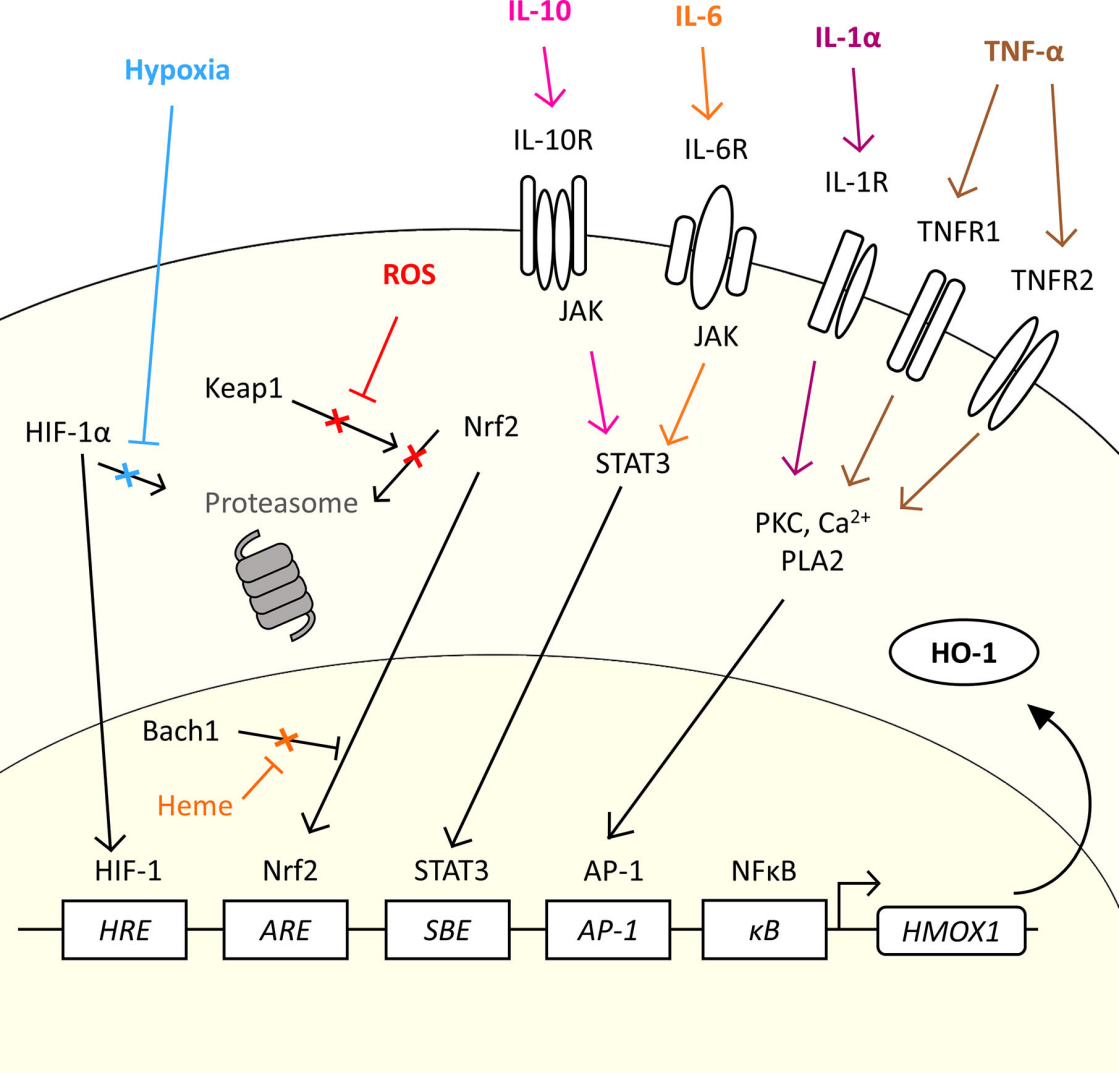
The diverse range of signals in the TME which could induce *HMOX1* expression. *HMOX1* mRNA expression is induced by a range of molecular and physical signals. The figure highlights common physical characteristics (hypoxia), metabolic by-products (ROS) and cytokines commonly associated with the TME which could induce the expression of HO-1 in cancer. In normal conditions, the transcription factor Nrf2 is inhibited by Keap1 which prevents nuclear translocation and promotes its proteasomal degradation. ROS generated by oxidative stress inhibit the interaction between Keap1 and Nrf2, allowing Nrf2 to translocate to the nucleus and bind to the *ARE* of the *HMOX1* gene. In the presence of heme, Bach1 is prevented from inhibiting Nrf2 access to the *ARE* allowing the expression of *HMOX1*. Under conditions of hypoxia, the transcription factor HIF-1α escapes proteasomal degradation and translocates into the nucleus where it forms a complex with HIF-1β and binds to *HRE* to up-regulate *HMOX1* gene expression. Cytokines such as IL-10 and IL-6 can induce expression *via* the JAK/STAT3 pathway, while IL-1α and TNF-α induced *HMOX1* expression through a PKC, Ca^2+^ signaling, and PLA2 dependent pathway and *AP-1* element in the promoter region.

The gene for HO-1, *HMOX1*, is regulated by several transcription factors which allow HO-1 to be expressed in response to a variety of stimuli which are relevant to the TME (20, 53, 59, 60) (**Figure 2**). Induction of HO-1 protein expression is a cytoprotective measure in both cancer and normal tissue in response to an increase in reactive oxygen species (ROS) levels (61). Tumor cells are under constant oxidative stress, generating high levels of ROS (62). Nuclear factor erythroid 2-related factor 2 (Nrf2) is a major transcription factor for the *HMOX1* gene. Nrf2 controls over 200 genes associated with the antioxidant response, which includes HO-1 (63), and has been considered as a therapeutic target in cancer, and even proposed as an oncogene (63). Nrf2-regulated *HMOX1* gene transcription is, however, tightly regulated. Under basal redox conditions, Keap1, a repressor protein, binds to the Nrf2 transcription factor preventing its nuclear translocation and activity, through promoting its ubiquitination and proteasomal degradation (64, 65). However, under conditions of oxidative stress, Keap1 undergoes oxidation of its sulfhydryl groups, leading to the release of Nrf2 which permits stabilization and nuclear translocation (66) (**Figure 2**). In the nucleus, Nrf2 hetero-dimerizes with small Maf (sMaf) proteins to mediate *HMOX1* expression through binding the antioxidant response element (*ARE*) site in the promoter region of the gene. However, access to the *ARE* site is further regulated by Bach1, a transcriptional repressor, which also hetero-dimerizes with sMaf proteins to compete for *ARE* elements, sterically preventing the access of Nrf2 to the site (67). Cellular heme levels regulate *HMOX1* transcription through directly binding to Bach1, causing its release from the DNA (68), which in turn promotes Nrf2 access to the *ARE* to drive the expression of *HMOX1* (69). Heme levels have been demonstrated to be increased in cancer (70, 71). As such, cellular heme exquisitely regulates the expression of HO-1 in a substrate-dependent manner. Several cellular stimuli activate the Nrf2 pathway, and Nrf2-dependent HO-1 activation, such as sphingosine-1-phosphate (S1P) which is released from apoptotic cells within the TME which engages with the sphingosine-1-phosphate receptor (S1PR), a G protein-coupled receptor (53).

There are other transcription factor binding elements with the promoter region which have been demonstrated to regulate *HMOX1* gene expression, including a hypoxia-responsive element (*HRE*) which make HO-1 part of the cellular response to hypoxia. Hypoxia is a common characteristic of the TME where proliferation of the malignant tumor cells outstrips angiogenesis leading to inefficient vascular networks and poor perfusion of the tumor tissue (72, 73). Hypoxia, stabilizes the transcription factor HIF-1α in the cytoplasm allowing it to escape proteasomal degradation and permitting its translocation to the nucleus where it complexes with HIF-1β and initiates expression of the *HMOX1* gene (74, 75) (**Figure 2**). In addition, the *HMOX1* promoter contains several transcription factor binding sites that allow a variety of both pro-and anti-inflammatory cytokines to regulate *HMOX1* gene expression (**Figure 2**), allowing HO-1 to play an important role in both the response and resolution phase of inflammation. The tumor stroma is a rich source of cytokines, soluble proteins which act as signaling molecules between cells, creating a complex crosstalk within the stroma and tumor cells (76). The cytokine milieu is different between cancers (27, 77), which contributes to the heterogeneity of the TME through influencing recruitment and/or polarization of cells such as is observed with TAM (27) and Treg (78) populations. Specifically in relation to HO-1 expression, IL-1α and TNF-α, which have pro-inflammatory effects, have been demonstrated to induce *HMOX1* gene expression through a pathway involving protein kinase C (PKC), Ca^2+^ signaling and phospholipase A2 (PLA2) (79), which directs *HMOX1* expression through the transcription factor AP-1 binding the *AP-1* binding site in the *HMOX1* promoter (80). The *HMOX1* promoter also contains a NF-κB binding element (81) which further facilitates *HMOX1* to be induced in response to pro-inflammatory signals. Anti-inflammatory cytokines also induce *HMOX1* expression, including IL-6 (27, 60) and IL-10 (82) *via* the JAK/STAT3 pathway and the STAT binding element (*SBE*) located in the promoter region of the *HMOX1* gene (60). The diversity of stimuli which induce *HMOX1* gene expression highlight the fundamental role of this enzyme within inflammation and stress responses which are exploited in the TME to facilitate tumor progression.

Although, the expression of HO-1 is heavily influenced by the TME, there are instances where HO-1 can be constitutively expressed by tumor cells due to oncogenes driving the *HMOX1* promoter (12) or genetic mutations within the promoter itself (25). The human *HMOX1* gene promoter has a 5′-flanking region containing GT microsatellite repeats, which vary in number between individuals and influences basal *HMOX1* transcription and inducibility of the gene in response to stimuli (83, 84). Polymorphisms in the number of GT repeats in the *HMOX1* promoter have also been linked to a susceptibility to cancer (85), where longer GT repeats, which result in lower basal levels of HO-1 expression, increase the likelihood of the individual developing gastric, lung and oral squamous cancer (85). *HMOX1* mRNA can also be modulated post-transcriptionally by microRNA-378, a small non-coding RNA that specifically targets and destabilizes *HMOX1* mRNA, preventing its translation (86). The broad range of stimuli and genetic aberrations that lead to HO-1 in cancer highlight the importance of this enzyme in modulating the inflammatory response and cancer progression.

## The influence of HO-1 on cell fate

**Figure 3:**
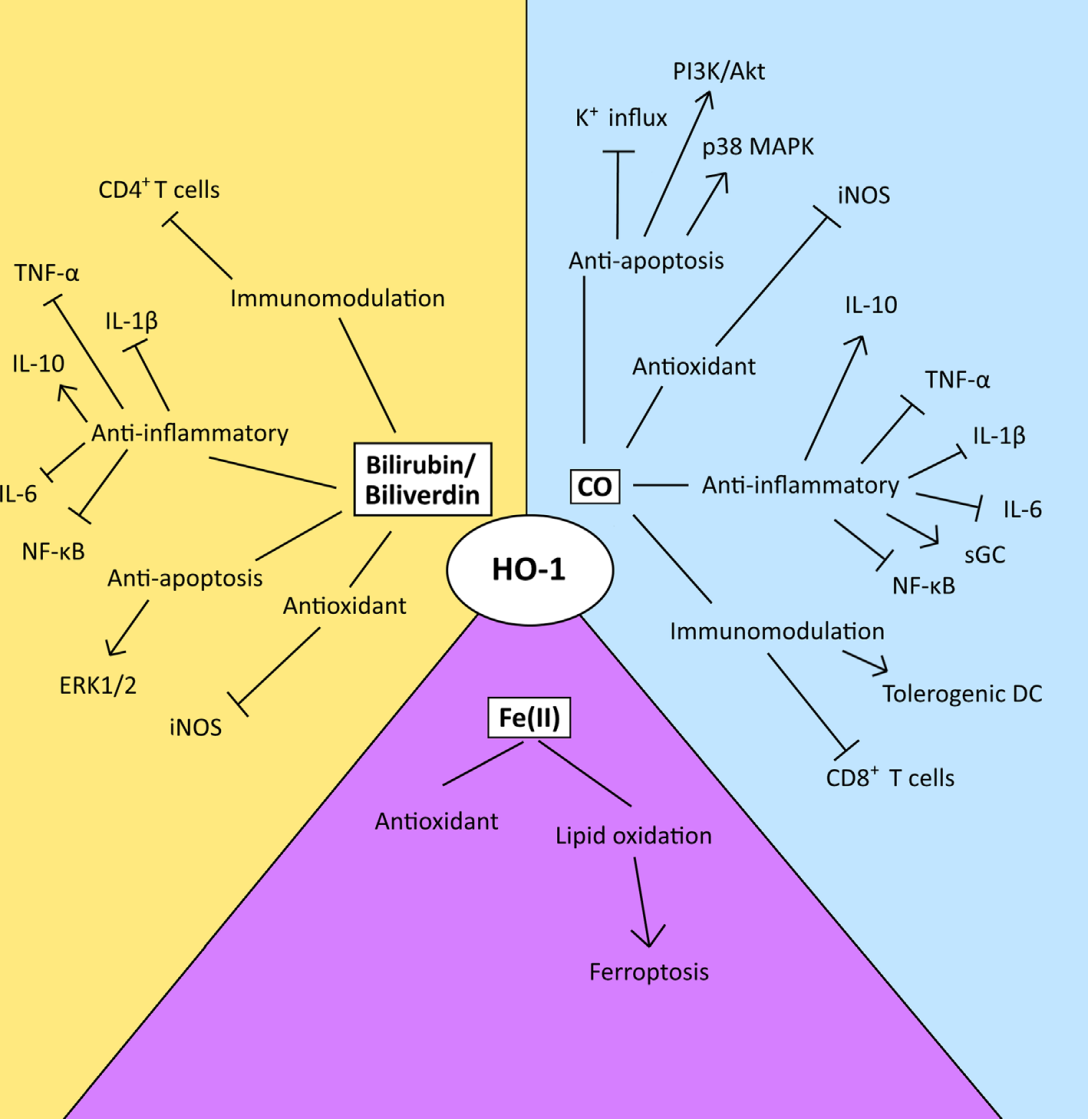
The diverse range of possible modulatory effects of the heme catabolites generated by the enzyme activity of HO-1 in the TME. This figure summarizes the possible modulatory effects of the heme catabolites in the TME. However, the relative utilization and biological importance of each pathway in disease progression or control is TME-dependent and key observations relating to these pathways are discussed within the text. CO and biliverdin/bilirubin mediate their anti-apoptotic effects via the modulation of signal transduction, and the inhibition of K^+^ influx. Their antioxidant effects are mediated via the inhibition of iNOS. Both CO and biliverdin/bilirubin inhibit the activity of pro-inflammatory cytokines, the NF-κB pathway and increase production of IL-10, and CO also activates sGC. CO induces the maturation of tolerogenic DCs and inhibits the activity and proliferation of CD8^+^ T-cells, while bilirubin inhibits the activation of CD4^+^ T-cells. Ferrous iron [Fe (II)] enacts antioxidant effects via ferritin, however, an imbalance between ROS levels and iron overload can lead to lipid oxidation and ferroptosis.

HO-1 is a well-established cytoprotective protein which confers its cytoprotective effects via the anti-apoptotic and antioxidant properties of the catabolites of heme degradation (42) (**Figure 3**). CO can inhibit the activity of pro-apoptotic K^+^ channels, as well as activate p38 MAPK and PI3K/Akt pathways to protect cells from apoptosis (28, 87, 88). CO generated from a HO-1-expressing endothelial cell line has also been demonstrated to be capable of conferring a survival signal to non-HO-1-expressing cells, highlighting the influence that CO can have on cell-cell communication (28). However, HO-1 activity has also been demonstrated to have an anti-proliferative/pro-apoptotic function in some contexts (89–91). Interestingly, CO’s role in dictating cell fate provides an interesting dichotomy in the context of the TME. For example, CO modulates the activity of a variety of kinases in the cell, in particular p38 MAPK, which can prevent apoptosis in endothelial cells (28) but conversely inhibits proliferation and enhances cell death in tumor cells (16, 92). The differences in response have been suggested to be a result of the underlying differences in the metabolism between malignant and healthy cells (56). Malignant cells fuel their proliferative capacity through adapting their metabolism to preferentially uptake and process glucose anaerobically, in a process known as the ‘Warburg’ effect (93). Treatment of tumor cells with CO has been demonstrated to result in their metabolic exhaustion and apoptosis due to switching their metabolic state to an increase in oxygen consumption in an ‘anti-Warburg’ type effect (16). This metabolic switch of the cancer cell to oxidative metabolism by CO has been demonstrated to decrease nucleotide and amino acid synthesis pathways, arrest the cell cycle and then ultimately lead to cell death due to the resulting intense mitochondrial stress and mitochondrial-dependent generation of ROS (16). Although a similar response has been observed in macrophages, the outcome is survival (94). Mechanistically, a role of CO in promoting DNA repair processes in healthy cells is also believed to contribute to outcome (28, 95). These observations have been exploited for therapeutic-gain, and supplying exogenous CO to mice bearing prostate or lung cancers, protects healthy tissue while targeting the malignant cells, resulting in tumor control in these models (16).

Although yet to be established in cancer, biliverdin/bilirubin has been demonstrated to also play a cytoprotective role, decreasing nitric oxide (NO) production by inhibiting iNOS expression (**Figure 3**) (96, 97) and mediating anti-apoptotic effects through the ERK1/2 pathway during hypoxia (98) in non-cancer models. Also, free chelatable Fe^2+^ can have a pro-oxidant role, by inducing lipid oxidation and ferroptosis. Ferroptosis is a form of non-apoptotic cell death that is caused by an overload of iron and is accompanied by lipid peroxidation (99) (**Figure 3**). Interestingly, experimental over-expression of the HO-1 protein can also cause a reversal of its cytoprotective role, as a result of the increase in reactive iron levels. Treatment with an iron-chelator significantly decreased signs of cellular injury including protein oxidation and lactate dehydrogenase (100). Ferritin is a protein which naturally sequesters free iron as a means to protects against oxidative stress (101). However, when HO-1 is highly expressed, the levels of ferritin become insufficient to neutralize the oxidative effects of reactive iron (102). The ferroptotic role of HO-1 has now been demonstrated in a range of tumor cells including in breast, lung and fibrosarcoma tumor cell lines (103, 104).

HO-1 expression is also induced by exposure to cytotoxic chemotherapies (CCTs) (105, 106) and provides a mechanism of resistance to CCTs such as; etoposide, doxorubicin, gemcitabine and cisplatin (45, 106–108), as well as other classes of anti-cancer drugs such as sapatinib, a pan-Her family kinase inhibitor (109). HO-1 can also indirectly affect the efficacy of CCTs through suppressing the immunomodulatory actions of these agents within the TME (2, 110). CCTs can both prime T-cell responses and elicit T-cell infiltration into the TME, which can turn immunologically ‘cold’ tumors ‘hot’ (2, 110). The immunomodulatory actions of CCTs are becoming increasingly understood and may actually underpin a significant proportion of their clinical efficacy (110–114). The immune suppressive actions of HO-1 activity within the TME (discussed below) can suppress CCT-elicited T-cell responses which indirectly effect the therapeutic efficacy of these agents (2). Interestingly, when tumor cells *in vitro* or tumor bearing mice *in vivo* are exposed to CO, CO can also play a contradictory role and increase the sensitivity of tumor cells to CCTs when exogenously delivered to the system, exacerbating the effect of CO-targeting mitochondrial function in malignant tumor cells to promote tumor cell death (16). In fact, PC3 prostate cancer cells exposed to exogenous CO became 1000-fold more sensitive to DNA damaging CCTs camptothecin and doxorubicin (16).

HO-1 activity has also been implicated as a tumor cell resistance mechanism for radiotherapy (115). The generation of ROS is a key event that underpins the anti-cancer effects of ionizing radiation (116), and pharmacological inhibition of HO-1 can enhance the radio-sensitivity of malignant cells (115, 117).

Interestingly, as these studies highlight, the role of HO-1 and cell fate is context and cell type-dependent which require careful considering in predicting and understanding how to most effectively exploit the HO-1 axis for therapeutic gain in the treatment of cancer.

## The Effect of HO-1 on Tumor Infiltrating T-Cells

**Figure 4:**
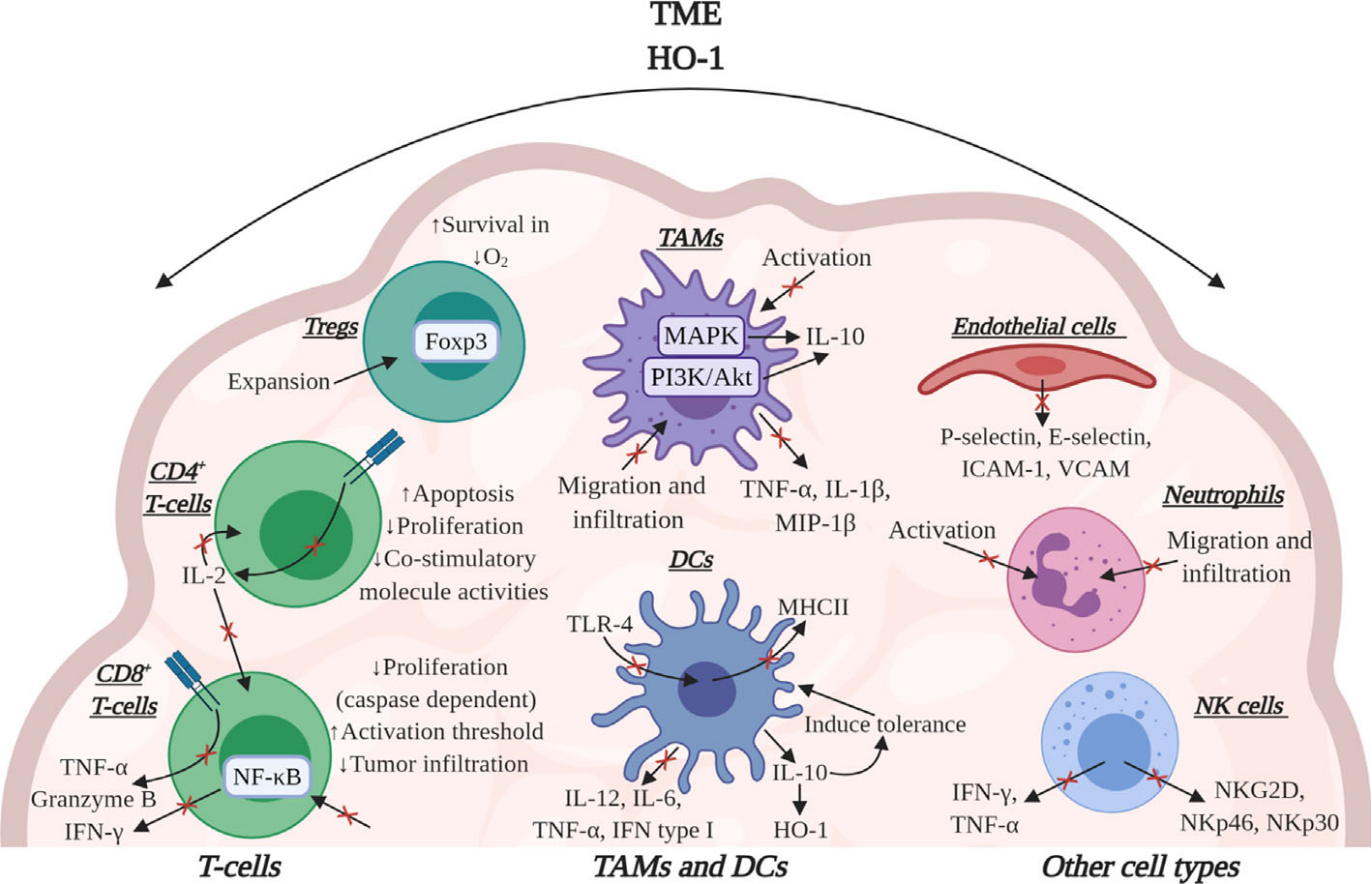
The effects of HO-1 activity on immune cells in the TME. HO-1 activity may affect several immune cell types to suppress the anti-tumor immune response. HO-1 reduces the effector functions of CD8^+^ T-cells by suppressing their expression of TNF-α, granzyme-B and IFN-γ. Specifically, CO suppresses the effector functions mediated via TCR engagement. HO-1 has also been implicated in suppressing CD8^+^ T-cell proliferation, tumor infiltration and increasing their activation threshold. In CD4^+^ T-cells CO can block TCR-dependent IL-2 production, thus influencing the proliferation of CD8^+^ and CD4^+^ T-cells. Bilirubin/Biliverdin can block CD4^+^ T-cell activity by inducing apoptosis, inhibiting proliferation and suppressing co-stimulatory molecule activities. In Tregs, HO-1 has been associated with the expansion of the population and HO-1-derived CO can improve Treg survival in conditions of hypoxia. HO-1 can also cause DCs to secrete IL-10 which maintains these cells in a tolerogenic state alongside up-regulating their expression of HO-1. HO-1 activity also down-regulates the expression of pro-inflammatory cytokines in these cells. Additionally, both CO and bilirubin can down-regulate MHCII expression in DCs. In TAMs, CO inhibits activation while also suppressing the release of pro-inflammatory cytokines and concurrently eliciting an increase in IL-10 production. In endothelial cells, CO can suppress the expression of adhesion molecules, affecting the ability of neutrophils to attach. HO-1 may also inhibit neutrophil activation itself and CO and biliverdin can inhibit migration and infiltration of both neutrophils and TAMs. Additionally, in NK cells HO-1 activity can decrease their cytotoxicity by suppressing their ability to secrete pro-inflammatory cytokines and express activatory cell receptors. Image was created using *BioRender* software.

HO-1 has potent immunomodulatory effects within the TME, influencing several cell types which underpin the anti-tumor immune response (**Figure 4**). Work in preclinical models of cancer have solidified the importance of HO-1, in some TMEs, to be pivotal to immune suppression which prevents efficient anti-tumor immunity as HO-1 activity suppresses T-cell effector function (2, 26). In mice bearing subcutaneous LL2 tumors which were rendered immunogenic through the expression of ovalbumin (LL2/OVA), the FAP^+^ TAM subset was the major tumoral source of HO-1. Specific conditional depletion of FAP^+^ TAMs, using diphtheria toxin in a bone marrow chimera of a FAP/diphtheria toxin receptor (DTR) transgenic mouse (54), permitted immunological control of tumor growth (26). Pharmacologically targeting HO activity using tin mesoporphyrin (SnMP) in mice bearing LL2/OVA tumors also permitted efficient immunological control of tumor growth (26). This study highlighted the biological significance of both FAP^+^ TAMs and their expression of HO-1 in tumoral immune suppression in the TME. In an aggressive spontaneous murine model of breast cancer (*MMTV-PyMT*), in which TAMs were also the major tumoral source of HO-1, an influx of T-cells into the TME were generated using the immune-stimulating CCT 5-fluorouracil (5-FU) which synergized with pharmacological inhibition of HO using SnMP to alleviate immune suppression and permit CD8^+^ T-cell dependent control of tumor growth (2). Although, small molecule inhibitors can have off target effects, SnMP which inhibits both HO-1 and HO-2, has no identified off-target effects to date (118–121). Furthermore, genetic inactivation of HO-1 in the myeloid lineage did not affect the prevalence of TAMs or CD8^+^ T-cells in the TME, but did improve the proportion of cytotoxic T-cells capable of producing the effector molecules granzyme-B, IFN-γ and TNF-α (2) (**Figure 4**). Due to poor infiltration of T-cells in these *MMTV-PyMT* tumors, genetic inactivation of *Hmox1* in TAMs neither affected the latency of tumor onset nor the kinetics of tumor growth, however, when 5-FU was administered to elicit a T-cell influx into the TME, tumor control was achieved (27).

The role of HO-1 expressing TAMs in immune suppression was further supported by a study using immunogenic OVA-expressing murine thymic lymphoma cells (EG7-OVA) (52). In this study, myeloid-specific HO-1 inactivation in mice increased proliferation, tumor infiltration and cytotoxic activity of CD8^+^ T-cells (52). CO has been demonstrated *in vitro* to be capable of modulating CD8^+^ T-cell effector function (26). This is likely achieved through CO’s ability to modulate cellular signaling, such as STAT1/3 (87) and NF-κB (122), which has the potential to directly compromise the anti-tumor response. NF-κB in particular, has been demonstrated to be vital for CD8^+^ T-cell effector function (123). However, it should be noted that bilirubin/biliverdin have also been demonstrated in non-cancer settings to be capable of suppressing NF-κB signaling (124–127).

Bilirubin has been demonstrated to block CD4^+^ T-cell activity by inducing apoptosis, inhibiting proliferation and suppressing co-stimulatory molecule activities (126). The immune suppressive actions mediated by human CD4^+^ CD25^+^ Foxp3^+^ Tregs have also been linked to HO-1 activity (58), however, the extent that HO-1 contributes to the immune suppressive actions of these cells remains debated in the literature (57, 128), and requires further investigation specifically in the context of cancer. CO has been demonstrated to improve Treg survival in hypoxic regions of the TME (129). Loss of HO-1 activity is associated with a loss of Tregs, whereas over-expression of HO-1 results in their accumulation (130) (**Figure 4**). Specifically, Tregs were demonstrated to induce tolerance in allografts when supplied with bilirubin or CO (131). In an elegant study which placed tumor bearing mice on a fasting mimicking-diet (FMD), the authors observed lowered HO-1 expression by the tumor cells, which rendered tumors more sensitive to doxorubicin and cyclophosphamide in the 4T1 model of breast cancer, and to doxorubicin in the B16 melanoma model, which resulted in CD8^+^ T-cell mediated tumor control associated with a loss of Tregs (130).

Several *in vitro* studies have demonstrated that HO-1 activity has the potential to suppress the expansion of T-cell populations (128, 132, 133). CO facilitates this through blocking CD4^+^ T-cell production of IL-2, a cytokine required for T-cell entry to the cell cycle (132) (**Figure 4**). In CD8^+^ T-cells, CO suppresses the expression and activity of caspase-3 and -8 through an up-regulation of a cyclin-dependent kinase inhibitor, p21^cip1^, a potent inhibitor against cell cycle progression (133). Interestingly, T-cells become insensitive to CO post-TCR signaling, highlighting the importance of HO-1/CO in facilitating a tolerogenic state in tissues through increasing the threshold for activation. The role of HO-1 activity in suppressing T-cell proliferation is also not just restricted to effector T-cell populations but also has been associated with the expansion of the immunomodulatory Treg population (128).

The pivotal role of HO-1 in modulating anti-tumor immune responses supports the consideration of HO-1 representing an innate immune checkpoint (2). Immune checkpoints are a family of regulatory proteins that act to suppress immune responses and T-cell activation/activity which are exploited in cancer to facilitate immune evasion. Therapeutically blocking the signaling of immune checkpoint molecules PD-1 and CTLA-4 has delivered unprecedented clinical responses in patients with cancer (134). Combining these therapies also improves the overall clinical response compared to the monotherapies (135), highlighting the redundency within this family of receptors. In a side-by-side comparison using 5-FU to elicit an anti-tumor immune response in *MMTV-PyMT* tumors, pharmacological inhibition of HO activity using SnMP displayed superior immunological control of tumor growth when compared to anti-PD-1 neutralizing antibodies (2). This observation demonstrated that, in some TMEs, HO-1 could be hierachically more important as an immune checkpoint than the clinically targeted PD-1, highlighting HO-1 as a potentially immportant immunotherapy target.

## The Broad Immunomodulatory Roles of HO-1 in the TME

HO-1 has been demonstrated to influence a variety of cell responses which contribute to tumor progression and enforce the immune suppressive and tissue protective characteristics of the TME. The catabolites of HO-1 activity can interfere with the phenotype, activation states and cytokine profile of several stromal cell populations which facilitates immune evasion by the tumor (42). DCs are important in activating the inflammatory response and developing T-cell immune responses against cancer (136) (**Figure 4**). The expression of HO-1 inhibits the maturation of pro-inflammatory DCs and ROS production maintaining DCs in a tolerogenic state (29, 30). This is mediated through a direct effect of CO on these cells (30), as well as indirectly through modulating their cytokine profile, decreasing the expression of IL-12, IL-6, TNF-α and type I IFNs while up-regulating IL-10 production (29, 30) (**Figure 4**). IL-10 has been demonstrated to block DC maturation (15) and can induce HO-1 protein expression (82, 137), creating a positive feedback loop. Both CO and bilirubin have been demonstrated to also down-regulate MHCII expression in DCs (30, 126, 138) which compromises the ability of these cells to present antigens to CD4^+^ T-cells (**Figure 4**). Toll-like receptor-4 (TLR-4) signaling is an important maturation signal for DCs. Endogenous (non-pathogen associated) TLR-4 ligands have been identified in the TME and have broadly become regarded as damage associated molecular patterns (DAMPs), such as high-mobility group box 1 (HMGB1) protein and heat shock protein 90 (110). Although the mechanism still needs to be studied in the cancer setting, CO has the potential to compromise the generation of anti-tumor immune responses through modulating TLR-4 signaling. CO can regulate the interaction of TLR-4 with caveolin-1, the principle structural protein of the plasmalemmal caveolae which regulates inflammatory signals from the cell membrane (139, 140) (**Figure 4**). Macrophage polarization can also be directly modulated by CO which has been demonstrated *in vitro* to contribute to skewing an anti-inflammatory phenotype of these cells (141). Macrophage exposure to CO can modulate their cytokine expression profile through an effect on MAPK signaling, down regulating pro-inflammatory cytokines such as TNF-α, IL-1β, and macrophage inflammatory protein 1-β (MIP-1β), while conversely upregulating IL-10 production (137) (**Figure 4**). Biliverdin has also been demonstrated to upregulate IL-10 production by macrophages through a PI3K-Akt dependent pathway (142). Furthermore, CO can suppress macrophage activation to a pro-inflammatory state through modulating TLR signaling, iNOS expression and the release of HMGB1, leading to the inhibition of macrophage activation (143, 144). However, there is also *in vivo* evidence using A549 tumors that exogenous CO exposure can polarize macrophages to a pro-inflammatory anti-tumor phenotype which are directly involved in facilitating a control of tumor growth (56).

Neutrophil and macrophage migration and infiltration can be inhibited by both CO and biliverdin which has been demonstrated in non-cancer inflammation and ischemia studies respectively (96, 145) (**Figure 4**). Studying the effect of CO in allograft rejection also revealed a decreased infiltration of macrophages and T-cells (145). This could be a result of a role of HO-1/CO on modulating endothelial cell activation (26), which can influence the attachment and extravasation event from the blood to the TME, and requires investigation in the context of cancer. CO has been demonstrated to suppress the activation of endothelial cells in response to cytokines *in vitro* and reduced their expression of key adhesion molecules P- and E-selectin, ICAM-1 and VCAM that prevented neutrophil adhesion (146) (**Figure 4**). HO-1 activity may also suppress neutrophil (147, 148) and natural killer (NK) cell (149, 150) activation and effector function (**Figure 4**). HO-1 activity has been reported to suppress NK cell activation through suppressing their expression of activatory receptors such as NKG2D, NKp46 and NKp30, as well as compromising their ability to secrete IFN-γ and TNF-α (149) (**Figure 4**). These intriguing observations highlight the need to further explore the role of HO-1 on neutrophils and NK cell function in the context of the TME. However, it is clear that HO-1 activity has the ability to influence the anti-tumor immune response both directly, indirectly and at different stages of its development, allowing HO-1 to be exploited by cancer to facilitate in immune evasion.

## HO-1 and Angiogenesis in Cancer

Neo-angiogenesis is pivotal to tumor progression and HO-1 has been demonstrated to modulate the process (42, 100, 151–153). *In vitro* models, not specific to cancer, have implicated a role for CO in promoting endothelial cell proliferation (154). Vascular endothelial growth factor (VEGF) is an important pro-angiogenic protein to cancer progression eliciting endothelial cell proliferation and vessel sprouting, and CO can act as a stimulus for inducing VEGF expression (155–157), which is mediated by a HIF-1α-dependent pathway (158, 159). However, there are conflicting reports in the literature that also suggest HO-1 over-expression can conversely elicit a down-regulation of VEGF and HIF-1α (92, 157), highlighting a potentially ‘tuning’ effect by CO that is dose dependent. Alongside these pro-proliferative roles of CO on vascular forming cells, CO has also been demonstrated to prevent endothelial cell apoptosis through NF-κB and p38 MAPK pathways and therefore indirectly influence angiogenesis, an effect which is reversed by inhibiting HO activity using SnMP (28, 137, 160). Pharmacological inhibition of HO-1 activity in LL2/OVA tumors also resulted in an elevation of tissue factor expression by endothelial cells in the TME. Tissue factor is a protein which initiates blood coagulation and is indicative of endothelial cell activation. This suggests that HO-1 activity may play a role in suppressing the activation state of these cells (26), which could influence the recruitment of immune cells to the TME (as discussed above). These diverse roles in which HO-1 activity has been implicated in facilitating angiogenesis provide an opportunity to target HO-1 as an anti-angiogenic strategy, however, it should be noted that in several preclinical models using pharmacological inhibition or genetic inactivation of HO-1 did not result in tumor control (2, 26, 27), suggesting that HO-1 could play a modulatory rather than non-redundant role in this process which may also be TME-dependent.

## HO-1 in Metastasis

Metastasis, the ability of tumor cells to colonize sites distal to that of the primary tumor, accounts for 90% of cancer-related deaths and there is an emerging role of HO-1 in the process. However, the role of HO-1 in relation to metastasis still remains debated and may rely on variables which have yet to be fully elucidated. There are several studies supporting the pro-metastatic role of HO-1, where over-expression of the enzyme has correlated with an increase in metastatic potential (152, 161–163). In human advanced colorectal cancer, HO-1 is correlated to lymph node metastasis and a shorter disease-free survival time (164). In a loss of function example, pharmacological inhibition of HO-1 activity using SnMP in mice bearing orthotopic 4T1 mammary tumors, despite not controlling growth of the primary tumor, suppressed the number of pulmonary metastasis (27). Interestingly, SnMP did not affect the pulmonary seeding of intravenously injected 4T1 cells suggesting that the mechanism of action was potentially occurring at the primary tumor site. *Ex vivo* studies demonstrated that HO-1 activity and CO were able to facilitate transendothelial migration of tumor cells, implicating a potential role for HO-1 in facilitating the intravasation event via a mechanism that was independent of vascular leakiness (27). Tumor cells are capable of adopting a more motile mesenchymal-like phenotype, referred to as epithelial to mesenchymal transition (EMT), which facilitates the metastatic potential of the cells. In a model of human glioma, CO was demonstrated to be capable of increasing tumor cell migration (165). There is also direct evidence that HO-1 activity can contribute to EMT (166). In xenograft models of PC3 in which HO-1 was inactivated in the TAMs, E-cadherin expression was inhibited, accompanied by the up-regulation of mesenchymal markers Twist-1 and Snail in the tumor cells, indicating evidence of EMT (51). Matrix metalloproteinases (MMPs), are a family of matrix degrading proteases required to breakdown the extracellular matrix to allow tumor cell migration through the tissue. In an anti-metastatic role of the HO-1 axis, CO has also been demonstrated to down-regulate the expression of MMP9 to conversely decrease metastasis in models of breast cancer (86, 167).

Several studies also highlight a role for HO-1 within the metastatic niche in the lung, which represent sites to which tumor cells preferentially colonize. Myeloid expression of HO-1 in the lung has been demonstrated to promote lung metastatic colonization *in vivo* (168). In agreement, studies using intravenously injected B16 melanoma cells also observed an increase in lung colonization when HO-1 was over-expressed (152, 169). Further investigation is required in relation to the role of HO-1 and its role in metastasis and the metastatic niche, to elucidate the biological rules which predict the outcome of HO-1 activity in the TME.

## Summary

Due to the breadth of the tumor promoting roles of HO-1, it is unsurprising that HO-1 is so widely expressed in cancer. These pathways are enacted by the biologically active catabolites of heme degradation to promote progression of the disease through anti-apoptotic, -oxidant, -inflammatory effects alongside pro-angiogenic and -metastatic effects. However, there are examples in the literature where HO-1 can play a converse anti-tumoral role. These contradicting roles of HO-1 highlight the complexity of the HO-1 axis in cancer. It is clear that in some cases the biological landscape of the specific TME may play a role in dictating the overall outcome of pharmacologically targeting HO-1 which can be dependent on the quality of the anti-tumor immune response and the degree of T-cell infiltration into the TME (2, 26). As the metabolic state of a cell can dictate the response to CO, such as is observed by its pro-apoptotic effects in malignant cells and anti-apoptotic effects in healthy cells (16). Also, the observation that in malignant tumor cells, HO-1 can become truncated and localized to the nucleus to elicit a transcriptional-regulatory role provides an intriguing additional layer to the functionality of this protein, but highlights the need to consider the cellular localization of HO-1 rather than just its presence or absence in the TME (11, 16, 23, 44). Also, there is scope for further studying the role of HO-1 expression level (92, 157) or catabolite concentration in dictating the response outcome in cancer (170, 171). Further *in vitro* investigations of the biological effects of endogenous cell derived heme catabolites produced by HO-1-expressing cells communicating to cells which do not express HO-1 is required to supplement the insight gained from exogenously supplied catabolites in such systems which could be at supra-physiological concentrations. In relation to exploiting our knowledge of the heme catabolites and HO-1 for therapeutic gain, the preclinical evidence demonstrating that exogenous CO exposure can deliver anti-tumor control provides a compelling translational avenue (16). In the context of boosting anti-tumor immunity, pharmacologically targeting HO activity as an immunotherapy approach may be equally attractive (2). However, no doubt as our understanding of the complexity of the TME increases, further roles for HO-1 will emerge in the years ahead.

## Author Contributions

KNLH, JEA, and JNA wrote the manuscript. All authors contributed to the article and approved the submitted version.

## Conflict of Interest

The authors declare that the research was conducted in the absence of any commercial or financial relationships that could be construed as a potential conflict of interest.
